# Activation of P2X_7_ Receptor by ATP Plays an Important Role in Regulating Inflammatory Responses during Acute Viral Infection

**DOI:** 10.1371/journal.pone.0035812

**Published:** 2012-04-25

**Authors:** Benjamin H. Lee, David M. Hwang, Nades Palaniyar, Sergio Grinstein, Dana J. Philpott, Jim Hu

**Affiliations:** 1 Research Institute, The Hospital for Sick Children, Toronto, Canada; 2 Toronto General Hospital/University Health Network, Toronto, Canada; 3 The Institute of Medical Science, University of Toronto, Toronto, Canada; 4 Department of Laboratory Medicine and Pathology, University of Toronto, Toronto, Canada; 5 Department of Biochemistry, University of Toronto, Toronto, Canada; 6 Department of Immunology, University of Toronto, Toronto, Canada; University of California Merced, United States of America

## Abstract

Acute viral infection causes damages to the host due to uncontrolled viral replication but even replication deficient viral vectors can induce systemic inflammatory responses. Indeed, overactive host innate immune responses to viral vectors have led to devastating consequences. Macrophages are important innate immune cells that recognize viruses and induce inflammatory responses at the early stage of infection. However, tissue resident macrophages are not easily activated by the mere presence of virus suggesting that their activation requires additional signals from other cells in the tissue in order to trigger inflammatory responses. Previously, we have shown that the cross-talk between epithelial cells and macrophages generates synergistic inflammatory responses during adenoviral vector infection. Here, we investigated whether ATP is involved in the activation of macrophages to induce inflammatory responses during an acute adenoviral infection. Using a macrophage-epithelial cell co-culture system we demonstrated that ATP signaling through P2X_7_ receptor (P2X_7_R) is required for induction of inflammatory mediators. We also showed that ATP-P2X_7_R signaling regulates inflammasome activation as inhibition or deficiency of P2X_7_R as well as caspase-1 significantly reduced IL-1β secretion. Furthermore, we found that intranasal administration of replication deficient adenoviral vectors in mice caused a high mortality in wild-type mice with symptoms of acute respiratory distress syndrome but the mice deficient in P2X_7_R or caspase-1 showed increased survival. In addition, wild-type mice treated with apyrase or inhibitors of P2X_7_R or caspase-1 showed higher rates of survival. The improved survival in the P2X_7_R deficient mice correlated with diminished levels of IL-1β and IL-6 and reduced neutrophil infiltration in the early phase of infection. These results indicate that ATP, released during viral infection, is an important inflammatory regulator that activates the inflammasome pathway and regulates inflammatory responses.

## Introduction

Acute viral infection poses serious health problems as seen in the recent outbreaks caused by the new strains of influenza virus and the SARS corona virus. Although the rapid viral replication and its cytopathic effects can directly damage the infected tissue, the overwhelming host response to acute viral infection can lead to a fatal outcome due to systemic inflammation and multiple organ failure [1], [2]. For this reason, the innate immune response is referred to as a double-edged sword as it is essential for inducing immune responses against pathogens but its over-activation can lead to immunopathologic consequences [3], [4].

Adenovirus is a double stranded DNA virus that can infect various organs in humans and often causes acute upper respiratory tract infection with relatively mild symptoms. Adenovirus has been engineered as replication deficient viral vectors for gene therapy purposes and shown to be one of the most effective gene delivery vehicles for the lung [5]. Although adenoviral vectors (Ads) have been used in gene therapy with mild side effects, a fatality occurred during a clinical trial. The patient suffered from systemic inflammatory response syndrome with pathological features of acute respiratory distress syndrome (ARDS) after receiving a high dose Ad [6]. The fact that administration of replication deficient Ads can induce severe inflammatory responses supports the notion that an overactive innate immune response is responsible for devastating consequences in the host during acute viral infection.

Studies on influenza virus demonstrated that NLRP3 inflammasome activation is a critical component of the innate immune response against acute viral infection [7], [8], [9]. It has been shown that DNA viruses such as adenovirus can also activate NLRP3 and AIM-2 inflammasome to induce secretion of IL-1β and IL-18 [10], [11]. Although the pathogen recognition receptors (PRRs) such as NLRP3 and AIM-2 are essential components of inflammasomes it has been recognized that additional stimuli are necessary for activation of the inflammasome pathway [12], [13], [14]. In studies examining the mechanism of inflammasome activation, ATP is often applied to induce secretion of IL-1β from macrophages following stimulation with pathogens or the relevant pathogen associated molecular patterns (PAMPs). ATP has been considered as an endogenous danger signal since cells maintain a high concentration of ATP but it is mostly absent outside of the cell [15]. In addition, P2X_7_ receptor (P2X_7_R), the cell surface receptor for ATP in macrophages and other immune cells, has an unusually low affinity raising speculation that the release of high concentration ATP from stressed or dying cells might provide an important regulatory mechanism for induction of inflammatory responses [16]. In most of the studies, ATP is exogenously added at milli-molar concentrations to stimulate P2X_7_R. Other studies utilized strong cytotoxic treatments or directly applying necrotic cells to show release of ATP form dying cell can induce inflammatory responses [17], [18]. While these studies indicate that ATP is involved in inflammatory responses, the role of ATP in regulating innate immune response during viral infection is still not clear.

In this study, we investigated whether ATP plays a role in induction of inflammatory responses during acute viral infection using replication deficient Ads. Replication deficient viral vectors can be a useful tool for studying the innate immune response against viral infection. Unlike wild-type virus models where the host is infected with a small number of viruses which then proliferate over time to reach the level of acute viral infection, a replication deficient viral vector can be administered at a pre-determined titer to emulate the condition of acute viral infection. Moreover, since there is no further viral replication that generates ongoing cytopathic effects, the infection models using replication deficient viruses allow us to examine the host induced innate immune responses. In this view, it should be noted that the mice with a deficiency in the innate immune system often do worse than wild-type mice when infected with wild-type viruses because the deficiency usually hinders induction of innate immune responses resulting in unchecked viral replication. However, the same deficient mice would be spared from the host-damaging effect that could be activated by the relevant innate immune mechanism when infected with non-replicating viruses.

Although many studies reported that administration of replication deficient Ad induced inflammatory responses their mechanisms have not been well characterized. However, studies clearly demonstrated that macrophages is a major player in Ad induced inflammatory responses as they uptake a large proportion of administered Ad and produce pro-inflammatory cytokines [19], [20], [21]. Using an *in vitro* model we have previously shown that Ad infection of macrophage and epithelial cell co-cultures produced substantially stronger inflammatory responses and increased cytotoxicity compared to infecting macrophages alone, suggesting synergistic interactions between these two cell types in regulating innate immune responses [22]. The study also showed that macrophage activation requires interaction with neighboring epithelial cells. Since macrophage activation often requires multiple stimuli [23], we postulated that the synergic responses are mediated by ATP released from Ad infected cells, which allows macrophage activation and triggers inflammatory responses. In this study, we found that ATP signaling via P2X_7_R plays a key role in regulation of inflammatory responses during acute viral infection including inflammasome activation. Furthermore, we show that ATP-mediated signaling is an important mechanism that regulates induction of systemic inflammation *in vivo*.

## Results

### Oxidized-ATP (oATP) inhibited inflammatory responses in the Ad infected macrophage and epithelial cell co-culture

**Figure 1:**
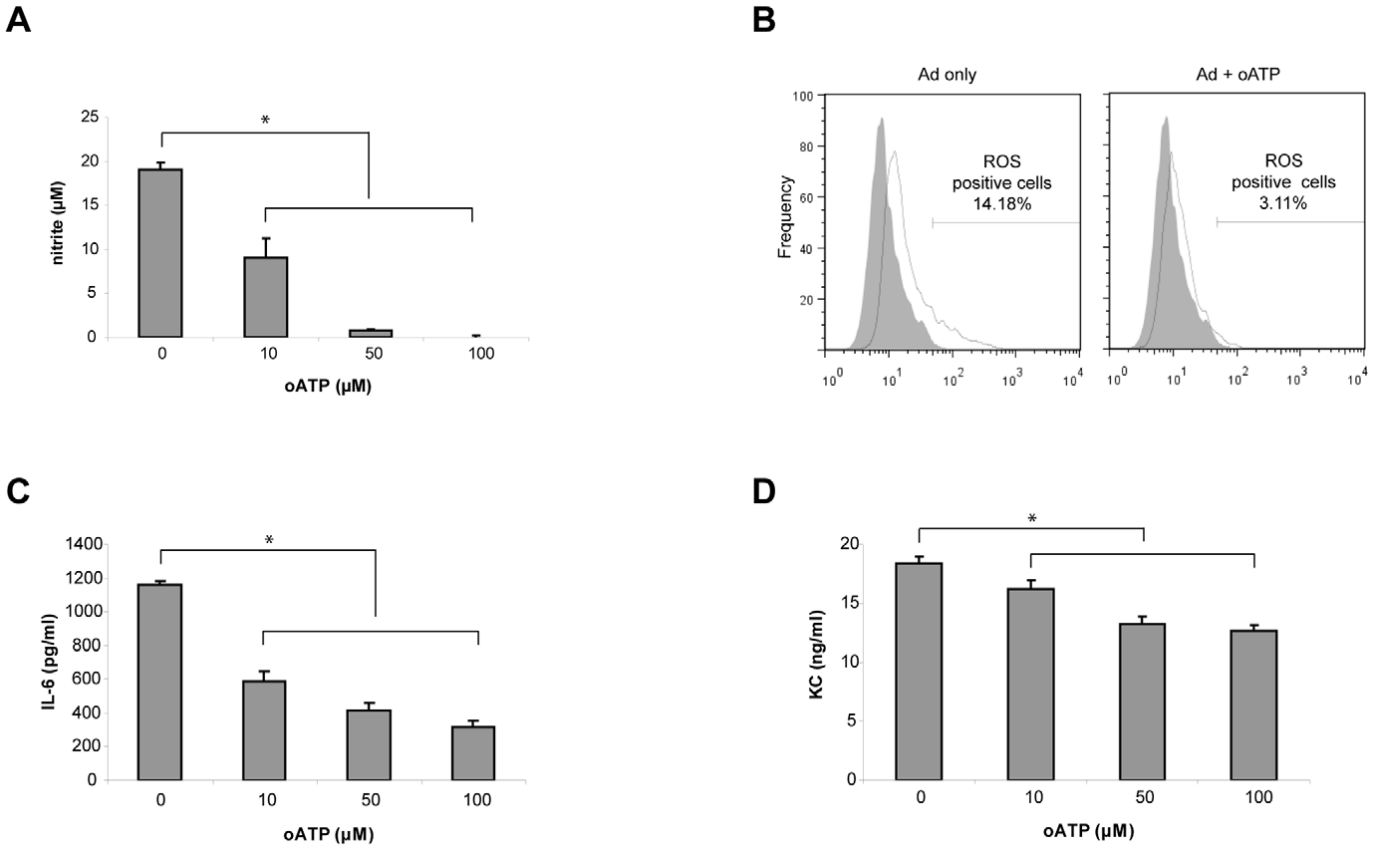
Treatment with oATP inhibited inflammatory responses in Ad infected MLE-Raw co-culture. The MLE-Raw co-culture was infected with Ad for 24 h in the presence of oATP at the designated concentrations. (A), The amount of NO produced in the culture was measured from the medium using Griess reagent. Data are expressed as mean ± SD. (B), The MLE-Raw co-culture was infected with Ad and ROS positive cells from were counted by flow cytometry after treating with 3-(p-aminophenyl) fluorescein (APF) without gating for specific cell types. Shown is a representative FACS histogram of three independent experiments with the percentage of ROS positive population. (C and D) Mouse IL-6 and KC, respectively, were measured from the medium using commercially available ELISA kits. Data are expressed as mean ± standard deviation (SD).

Previously, we found that Ad infection of the co-culture consisted of a mouse lung epithelial cell line, MLE-15 (MLE), and a mouse macrophage cell line, Raw 264.7 (Raw), resulted in induction of inflammatory mediators including pro-inflammatory cytokines, nitric oxide (NO), and reactive oxygen species (ROS) [22]. These inflammatory mediators were absent or much lower when Raw cell mono-culture was infected with Ad. In order to test whether ATP is involved in the synergistic response we first measured the concentration of ATP in the media after Ad infection. Although there was a variation in magnitude, we consistently observed higher extracellular ATP in the Ad infected co-culture 12∼18 h after infection, which coincides to the time when there is a significant increase in the cytotoxicity measured by LDH in the medium [22]. To further investigate the correlation between the increase in extracellular ATP and the inflammatory responses, we utilized oATP [24] to inhibit for ATP-mediated macrophage activation. Infection of the co-culture with approximately 20 multiplicity of infection (MOI) of Ad induced a significant amount of NO within 24 h. However, adding 50∼100 µM of oATP completely abolished NO generation in the Ad infected co-culture (Figure 1A). We also found that the treatment with 100 µM of oATP reduced the number of ROS positive cells to a basal level (Figure 1B). Furthermore, oATP treatment significantly decreased induction of key inflammatory cytokines such as IL-6 and KC (Figure 1C and D). These inhibitory effects of oATP on induction of various inflammatory mediators suggest that ATP plays a role as an intercellular signal molecule in the macrophage and epithelial cell co-culture during Ad infection.

### Deficiency in P2X_7_R inhibited inflammatory responses in Ad infected macrophage and epithelial cell co-culture

**Figure 2:**
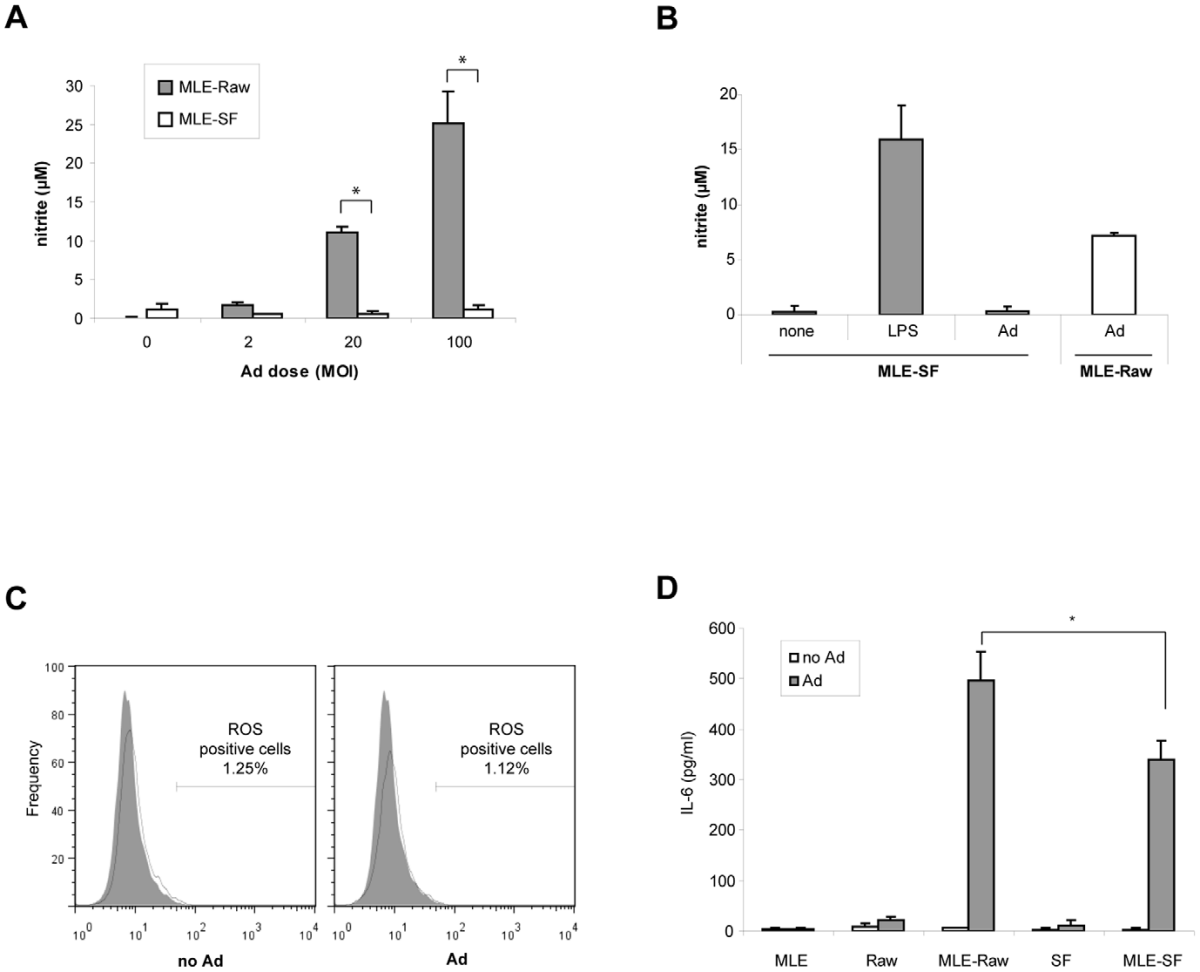
Ad infection of the co-culture with P2X_7_R deficient Raw cells generated significantly less inflammatory mediators. The co-culture of MLE and a P2X_7_R deficient Raw cell (SF) was established as MLE-Raw co-culture. (A) MLE-Raw and MLE-SF co-cultures were infected with the designated MOI of Ad. The amount of NO produced in the culture medium was measured by Griess assay. Data are expressed as mean ± SD. (B) To verify that SF cells has no inherent defect in NO generation MLE-SF co-culture was treated with LPS (2.5 µg/ml) and NO generation was compared with Ad infected MLE-SF as well as MLE-Raw co-cultures. Data are expressed as mean ± SD. (C) The ROS positive cells in the Ad infected MLE-SF co-culture were counted by flow cytometry analysis after treating cells with APF. Shown is a representative FACS histogram of three independent experiments with the percentage of ROS positive population. (D) Mouse IL-6 was measured from the media of mono-cultures (MLE, Raw, and SF) and co-cultures (MLE-Raw and MLE-SF) 24 h after Ad infection using a commercially available ELISA kit. Data are expressed as mean ± SD.

Despite the common usage of oATP to inhibit ATP-mediated inflammatory responses, there is a debate about whether its effect is caused by inhibition of cell surface ATP receptors or by inhibition of other signaling pathways [25]. Macrophages express several ATP receptors on the cell surface but P2X_7_R is known to play the major role in regulating inflammatory responses [26]. In order to confirm our findings from the experiments with oATP we performed co-culture experiments with a P2X_7_R deficient Raw cell line (SF) [27]. Unlike the co-culture with the wild-type Raw cells, Ad infection of the MLE-SF co-culture produced only basal level of NO (Figure 2A). Even at the five time higher Ad dose we did not detect any significant NO induction in the co-culture of P2X_7_R deficient Raw cells whereas LPS treatment induced a large amount of NO indicating that these cells are capable of NO generation (Figure 2B). In addition, Ad infection of the MLE-SF co-culture did not generate ROS positive cells (Figure 2C) and produced less IL-6 compared to its wild-type counterpart (Figure 2D), the results similar to the ones obtained from the experiments using oATP. These data indicate that P2X_7_R activation by extracellular ATP is an important component of the inflammatory responses in macrophages during Ad infection.

### MLE-Raw co-culture failed to secret IL-1β after Ad infection

While our study was in progress Pelegrin *et al*. reported that Raw cells do not express ASC (apoptotic speck protein containing a caspase recruitment domain), one of the key molecules of the inflammasome complex, and, therefore, they are deficient in IL-1β and IL-18 processing and secretion [28]. Indeed, we detected no IL-1β in the media of the Ad infected MLE-Raw co-culture although there was induction of IL-1β gene expression [22] and pro-IL-1β was detected from the cell extract (Figure S1). Therefore, we utilized another mouse macrophage cell line, J774.A1 (J774), in the co-culture system to investigate whether ATP is involved in the inflammasome activation and IL-1β secretion during Ad infection.

### Ad infection of MLE-J774 co-culture induced secretion of IL-1β and IL-18

**Figure 3:**
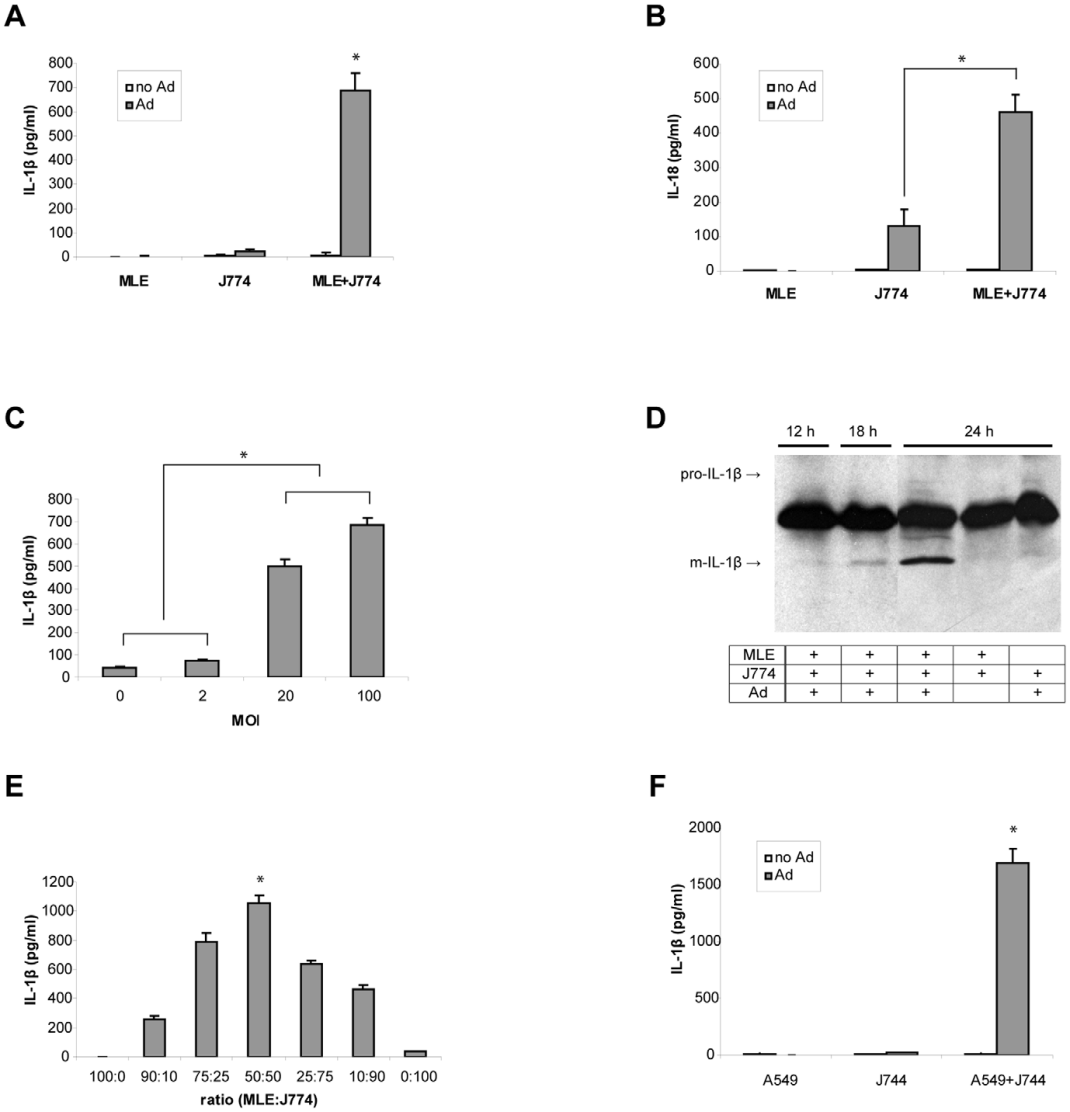
Ad infection of epithelial cell and macrophage co-culture induced secretion of IL-1β and IL-18. (A and B) MLE and J774 mono-culture as well as MLE-J774 co-culture were infected with Ad. IL-1β (A) and IL-18 (B) secretion was measured by ELISA assay using the media 24 h after infection. Data are expressed as mean ± SD. (C) MLE-J774 co-culture was infected with 0, 2, 20, or 100 MOI of Ad and IL-1β in the medium was measured 24 h after infection. Data are expressed as mean ± SD. (D) MLE-J774 co-culture was infected with Ad and medium was collected at designated time for Western blot analysis following immunoprecipitation. (E) Co-cultures of different ratio of MLE and J774 were established and IL-1β was measured in the medium 24 h after Ad infection. Data are expressed as mean ± SD. (F) A human lung epithelial cell line, A549, instead of MLE was used to establish the co-culture with J774 macrophages and infected with Ad. IL-1β was measured 24 h after infection. Data are expressed as mean ± SD.

Ad infection of MLE-J774 co-culture showed similar response to MLE-Raw co-culture including increase in cytotoxicity (Figure S2A) and induction of pro-inflammatory cytokines (Figure S2 B and C). In addition, MLE-J774 co-culture secreted a significant amount of IL-1β in the media within 24 h after Ad infection, which was absent in the J774 mono-culture (Figure 3A). We also detected a significant amount of IL-18 in the Ad infected co-culture (Figure 3B) suggesting that Ad infection of MLE-J774 co-culture induced activation of inflammasome. IL-1β secretion was more evident when infected with more than 20 MOI indicating that it requires above a threshold level of infection (Figure 3C). As shown by Western blot analysis the majority of IL-1β in the media was in the mature form and detected after 18 h (Figure 3D), corresponding to the time when drastic changes were observed in the cell culture. We found that the amount of IL-1β was maximized when the co-culture was consists of 50∶50 ratio of two cell types (Figure 3E). This result along with the fact that infection of J774 cells alone did not induce IL-1β secretion indicates that inflammasome activation requires additional signals generated from the interaction between neighboring macrophages and epithelial cells. Interestingly, the co-cultures consisted of mouse macrophage cells and human airway epithelial cells (A549) also induce IL-1β secretion, supporting the idea that inflammasome activation might be regulated by a biologically universal molecule such as ATP (Figure 3F).

### Inhibition of P2X_7_R and caspase-1 reduced IL-1β secretion in Ad infected co-culture

**Figure 4:**
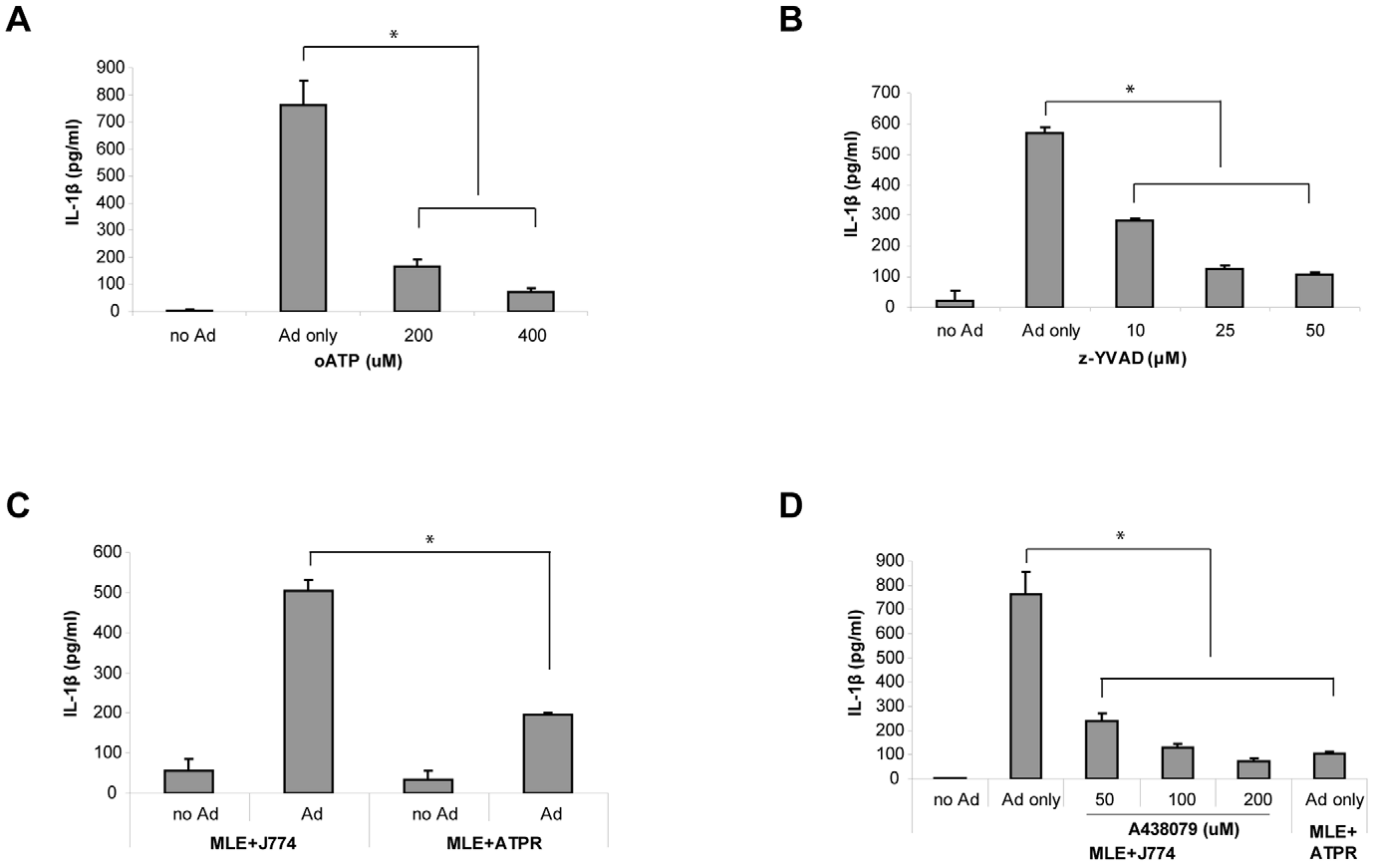
Inhibition of P2X_7_R and caspase-1 reduced IL-1β secretion in the Ad infected co-culture. (A and B) MLE-J774 co-culture was infected with Ad in the presence of oATP (0∼400 µM) or z-YVAD-fmk (0∼50 µM) and IL-1β secretion was measured in the medium. Data are expressed as mean ± SD. (C) The co-culture of MLE and a P2X_7_R deficient J774 cell line (ATPR) was establish and IL-1β in the medium was measured 24 h after Ad infection. Data are expressed as mean ± SD. (D) MLE-J774 co-culture was treated with a P2X_7_R specific inhibitor, A438079 (0∼200 µM) at the designated concentration before Ad infection and IL-1β was measured from the medium 24 h after infection. Data are expressed as mean ± SD.

To test whether ATP is involved in IL-1β secretion we treated the MLE-J774 co-culture with oATP. As shown in Figure 4A, 200–400 µM of oATP significantly reduced IL-1β secretion. IL-1β secretion was also inhibited by z-YVAD-fmk indicating that this process is caspase-1 dependent and involves inflammasome activation (Figure 4B). To address the role of P2X_7_R in inflammasome activation and IL-1β secretion during Ad infection we used a J774 cell line deficient in P2X_7_R (ATPR-B2 (ATPR)) [29]. Ad infection of MLE-ATPR co-culture produced significantly less IL-1β compared to MLE-J774 co-culture (Figure 4C). Furthermore, we observed similar results when MLE-J774 co-culture was treated with a P2X_7_R specific inhibitor [30], A-438079 (Figure 4D), suggesting that P2X_7_R activation by ATP is an important process in inflammasome activation and IL-1β secretion during Ad infection.

### Co-cultures consisted of primary macrophages with deficiency in P2X_7_R and caspase-1 secreted less IL-1β after Ad infection

**Figure 5:**
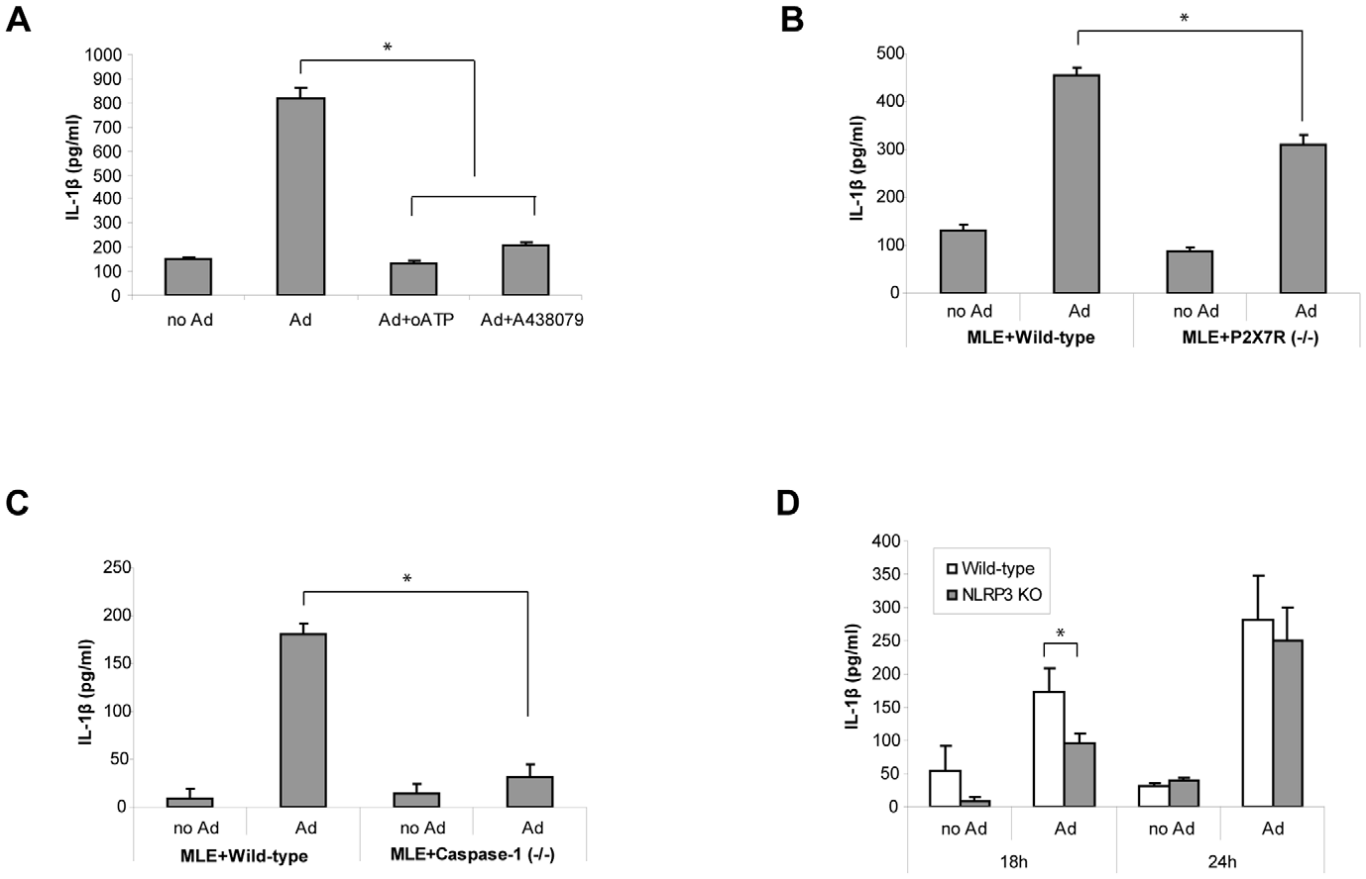
Co-cultures with primary macrophages from P2X_7_R and caspase-1 knockout mice secreted less IL-1β. (A) The co-culture was established with MLE and peritoneal macrophages obtained from wild-type (C56BL/6) mice and infected with Ad with or without oATP (200 µM) or A438079 (100 µM). IL-1β was measured from the medium 24 h after infection. Data are expressed as mean ± SD. (B–D), Peritoneal macrophages were collected from and P2X_7_R (B), caspase-1 (C), or NLRP3 (D) knockout mice and the co-cultures were established with MLE. IL-1β was measured from the medium 24 h (18 h and 24 h for NLRP3-KO) after Ad infection and compared to the wild-type counterpart. Data are expressed as mean ± SD.

We confirmed our results obtained with J774 macrophage cell line by replacing it with primary peritoneal macrophages obtained from mice. When infected with Ad the co-culture containing peritoneal macrophages from wild-type mice showed IL-1β secretion comparable to the co-culture with J774 cells. IL-1β secretion in the co-culture with peritoneal macrophage was significantly reduced when treated by P2X_7_R inhibitors (Figure 5A) in a similar manner as with the MLE-J774 co-culture (Figure 4). Furthermore, the co-cultures of peritoneal macrophages from P2X_7_R or caspase-1-knock out (KO) mice secreted significantly less IL-1β after Ad infection compared to the co-culture with peritoneal macrophages from wild-type mice (Figure 5B and C) demonstrating that Ad infection induced IL-1β secretion via inflammasome activation in mouse primary macrophages. We also performed the same experiment with the peritoneal macrophages from the NLRP3-KO mice since NLRP3 has been shown to be involved in Ad induced inflammation [10]. Although there was some reduction in IL-1β secretion in the NLRP3-KO macrophage co-culture the difference was less pronounced and limited to earlier time points (Figure 5D).

### Intranasal (i.n.) Ad infection in mice caused systemic inflammation and fatality but deficiency or inhibition of P2X_7_R and caspase-1 enhanced survival

**Figure 6:**
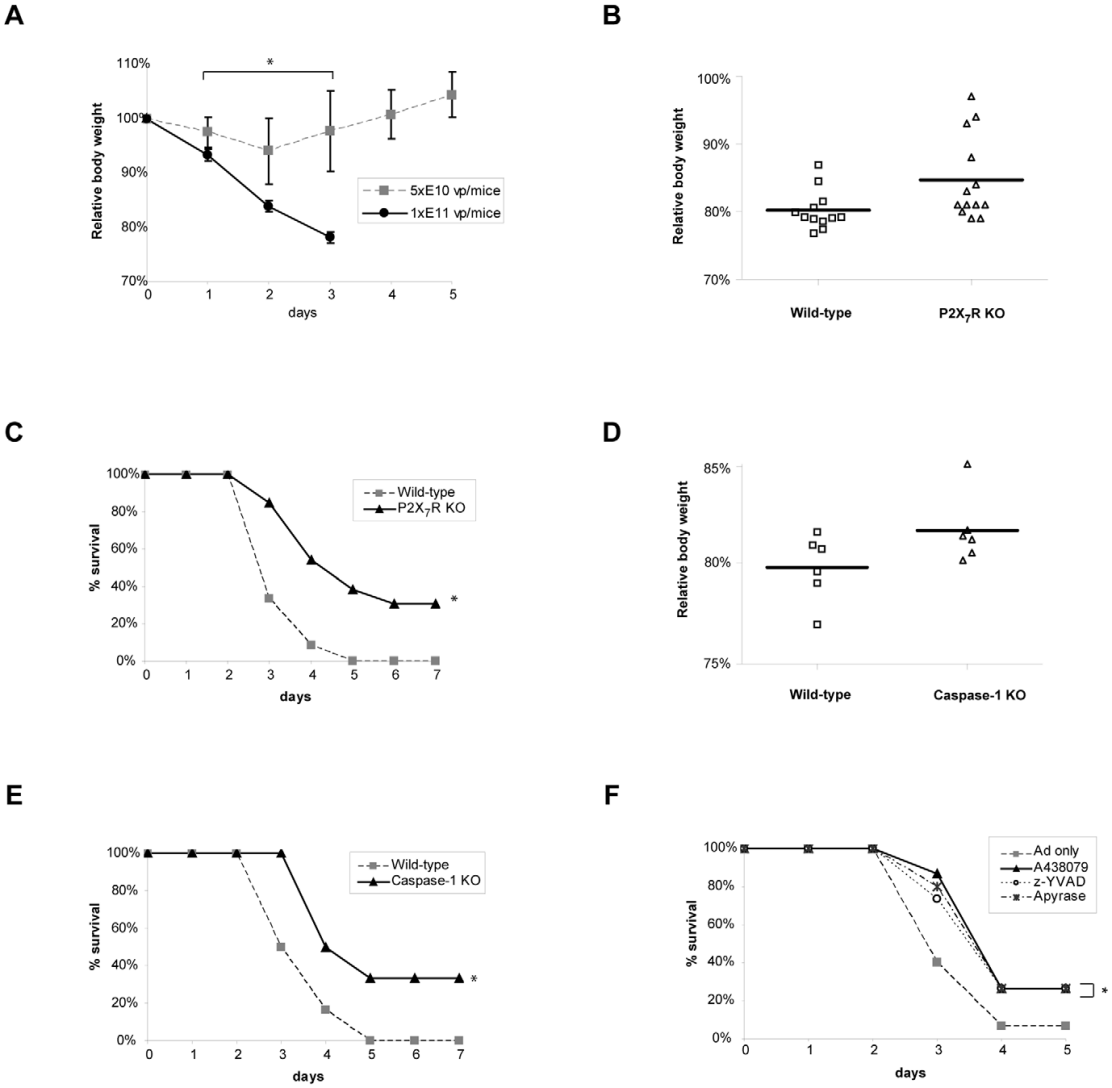
Intranasal Ad infection in mice caused ARDS and fatality but inhibition of P2X_7_R and caspase-1 enhanced survival. Ad was intranasally administered to mice with matching age and gender. The mice were monitored and body weight was measured each day. The survival curves were generated based on the humane end point of 20% weight loss and the comparison was made by the log rank test. (A) The wild-type (C57/B6) mice were infected with Ad at two dosages, 5×10^10^ or 1×10^11^ vp/mouse. The mice that received 5×10^10^ vp (n = 5) showed no apparent symptoms and sustained only slight weight loss but recovered within a few days. The mice that received 1×10^11^ vp (n = 5) showed ARDS like symptoms and continuously lost their body weight to reach the experimental humane end point. In the subsequent *in vivo* experiments Ad dosage of 1×10^11^ vp/mouse was used. (B and C) The wild-type (n = 12) and the P2X_7_R-KO mice (n = 13) were intranasally infected with Ad. The individual body weight on day 3, when all the subject mice were still alive, was presented as the relative retained body weight. The bold line represents the average body weight. (D and E) Similar to B and C, but the wild-type (n = 6) and the caspase-1-KO mice (n = 6) were compared. (F) The wild-type mice (n = 15/group) were treated with A438079 (300 µmol/kg), z-YVAD-fmk (10 mg/kg), or apyrase (4 U/mice) on day 0 and day 1of Ad administration and their survival curve was generated.

In order to study the role of ATP during acute Ad infection *in vivo* we established a mouse i.n. Ad infection model. Although Ad administration has been known to induce inflammatory responses, i.n. administration of Ad at dosages up to 5×10^10^ viral particles (vp)/mouse was well tolerated, showing only minor symptoms. On the other hand, when dosages higher than 1×10^11^ vp/mouse were given mice showed visible symptoms such as lethargy, dyspnea, ruffled fur, and significant and continuous weight loss followed by mortality within a few days (Figure 6A). These pathological features of systemic inflammation were very similar to ARDS described in other mouse models for acute pulmonary viral infection [1], [2]. In order to test whether inhibiting ATP-mediated inflammatory responses can alleviate the severity of symptoms caused by acute Ad infection we compared the weight loss and the survival rate between the wild-type and P2X_7_R-KO mice. As shown in Figure 6B, three days after infection most of the wild-type mice had considerable weight losses and showed symptoms of severe respiratory distress. However, the weight loss in P2X_7_R-KO mice was delayed and less severe (Figure 6B). More importantly, more than 30% of P2X_7_R-KO mice survived the infection (Figure 6C) and most of the surviving mice recovered the normal body weight and exhibited normal behavior by day 7. We found similar results from caspase-1-KO mice, which also exhibited less severe weight losses (Figure 6D) and a higher survival rate (Figure 6E) compared to the wild-type mice.

### Inhibition of ATP-P2X_7_R signaling enhanced survival of wild-type mice after Ad infection

To further support that the inhibition of ATP-P2X_7_R mediated inflammatory responses can alleviate acute inflammation and reduce fatality, we treated wild-type mice with A437980 [31], z-YVAD-fmk [32], or apyrase [33], a ATP hydrolyzing enzyme. These inhibitors were intraperitoneally administered twice, once at the time of Ad infection and once at 24 h after infection, in order to limit the effect of the inhibition to the initial stage of innate immune response. As shown in Figure 6F, the treatment with the inhibitors only during the first two days of infection significantly improved the survival in a similar manner as for the P2X_7_R and caspase-1-KO mice. These results from the inhibitor study along with the KO mouse studies demonstrate that ATP-P2X_7_R mediated signaling and subsequent activation of inflammasome pathway is critical for induction of systemic inflammation during acute viral infection *in vivo*.

### Enhanced survival in P2X_7_R-KO mice is due to reduced inflammatory responses

**Figure 7:**
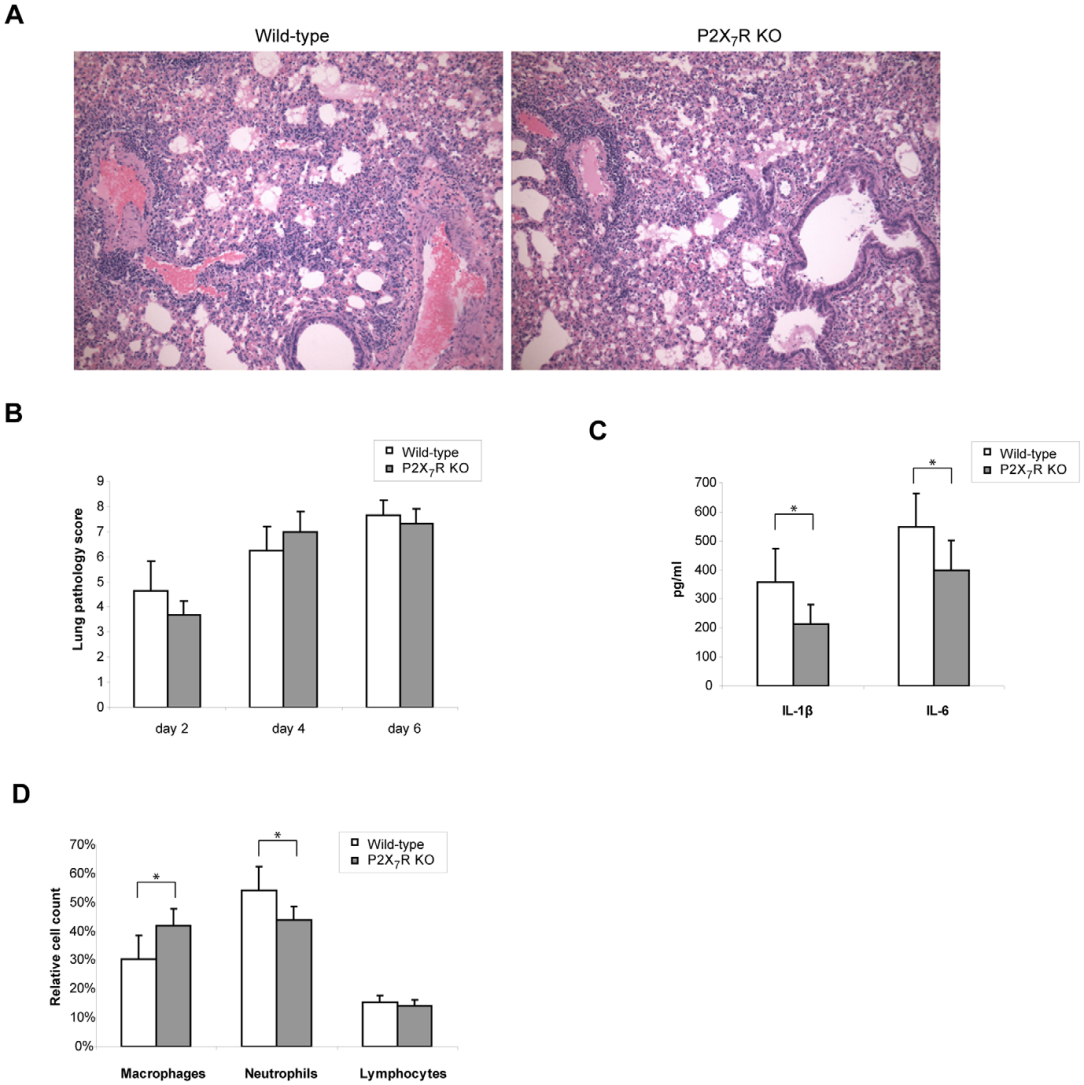
P2X_7_R-KO mice generated less inflammatory cytokines and neutrophil infiltration in the early stage of Ad infection. The wild-type (C57/B6) and the P2X_7_R-KO mice were infected with Ad (1×10^11^ vp/mice) by i.n. administration. (A) The mice were sacrificed on day 4 (n = 4) and the lungs were obtained for histological analysis. Representative microscopic sections are shown. See Figure S3 for sections from day 2 and 6. (B) The lung pathological scores were used to compare difference between the wild-type and the P2X_7_R-KO mice according to the criteria described in the Materials and Methods. Data are expressed as mean ± SD. (C and D) Twenty four hours after Ad administration BALF was collected from the wild-type (n = 10) and the P2X_7_R-KO mice (n = 8). IL-1β and IL-6 in the BALF were analyzed by ELISA (C). Differential cell counts were performed with the cell fraction of BALF (D). Data are expressed as mean ± SD.

Despite the apparent differences in the overall response to Ad infection the histopathologic features of the lungs in P2X_7_R-KO mice were similar to those of the wild-type mice (Figure 7A, B and Figure S3), suggesting that their survival advantage might be related to reduced host immune responses rather than the cytopathic damage caused by viral infection. We also found that there is no difference between wild-type and P2X_7_R-KO mice in the viral titers in the bronchoalveolar lavage fluid (BALF) 24 h after infection (data not shown) suggesting that the different phenotype was not due to the difference in viral clearance. It is well known from studies on acute viral infection as well as sepsis that overwhelming cytokine production and excessive neutrophil infiltration are the main immunopathological features linked to systemic inflammation and ARDS [34], [35]. Therefore, we measured the levels of pro-inflammatory cytokines, IL-1β and IL-6, in the lung 24 h after Ad infection. Both IL-1β and IL-6 in the BALF were significantly lower in the P2X_7_R-KO mice compared to the wild-type mice (Figure 7C). In addition, we found fewer neutrophils and more macrophages in the BALF of the P2X_7_R-KO mice compared to the wild-type mice at 24 h after infection (Figure 7D) indicating that neutrophil infiltration is delayed or attenuated. These attenuated responses in the P2X_7_R-KO mice compared to the wild-type C57/Bl6 mice underscore the significant role of P2X_7_R, especially when taking into consideration that the C57/Bl6 strain has been shown to be less responsive to its agonists such as ATP due to a point mutation in the cytoplamsic TNFR1 domain of P2X_7_R [36]. Taken together these results suggest that ATP signaling through P2X_7_R regulates induction of pro-inflammatory cytokine and neutrophil infiltration, which consequently lead to the host-damaging systemic inflammation during acute viral infection.

## Discussion

In this study we investigated the inflammatory responses against acute viral infection using replication deficient Ad to examine the effects of host mediated responses. Specifically, we focused on the role of ATP in induction of inflammatory responses. Using macrophage and epithelial cell co-culture systems, we showed that ATP signaling through P2X_7_R is essential for induction of inflammatory responses including activation of inflammasome during acute viral infection. Our co-culture system was an effective model for examining the role of ATP in inflammatory responses during viral infection without the use of exogenous ATP. Furthermore, our *in vivo* study demonstrates that the ATP-P2X_7_R signaling plays an important role in the development of systemic inflammation during acute viral infection.

Although a number of studies have examined the role of viral PAMPs and their relevant PRRs in inflammation, relatively little attention has been given to the role of endogenous danger signals in viral infection. This is partly due to the paucity of models that can be used to study the effect of danger signals. Infection with a wild-type virus inevitably accompanies proliferation of the pathogen and cell lysis that generate not only more PAMPs but also various danger signal molecules, making it difficult to dissect the effects of one from another. The replication deficient viral vector provides a unique model allowing us to generate the condition of acute viral infection without ongoing viral proliferation. In this model, cytopathic effects should be limited to the early stage of infection and the consequent inflammatory responses would be largely mediated by the innate immune mechanisms. Therefore, in the replication deficient viral infection model, the innate mechanisms triggered by endogenous danger signal should be more specifically revealed.

Although it was suggested more than a decade ago that ATP can modulate various immune responses, there is an increasing interest in the function of extracellular ATP with the recent discovery of the inflammasome pathway [37]. Stimulation of innate immune cells like macrophages with PAMPs followed by treatment of ATP has been commonly used to trigger inflammasome activation and IL-1β secretion in *in vitro* experiments. However, the role of ATP in inflammation and its physiological and pathological implications are only beginning to emerge. Using a bleomycin-induced lung injury model Riteau *et al.* have shown that ATP released from the injured cells is responsible for release of IL-1β and pulmonary inflammation [38]. Idzko and co-workers have examined the role of ATP in various inflammatory conditions from lung injury to asthma and graft-versus-host disease [33], [39], [40], [41]. Using experimental animal models their studies showed that inhibition or deficiency of P2X_7_R resulted in reduced severity in inflammation. Although these studies were conducted in models that cause tissue damages or immunogenic responses in the absence of pathogenic infection they provide important evidence that ATP can act as a potent inflammatory stimulator.

In our *in vivo* infection model, a high dose of replication deficient Ad is administered by intranasal route emulating the condition of acute viral infection. The infection resulted in ARDS-like symptoms and fatality demonstrating that a high titer of virus even without viral replication can trigger systemic inflammation in mice. Although it is not clear what causes systemic inflammation and ARDS, over production of cytokines has been proposed as a factor contributing to the severity of the disease in viral infection [34]. Our data support this idea as the decrease in pro-inflammatory cytokines, such as IL-1β and IL-6, in the P2X_7_R-KO mice correlated with reduced severity in inflammatory symptoms and higher survival rates (Figure 7C). IL-1β has been considered one of the key cytokines involved in the pathology caused by acute inflammation [42]. In particular, studies on influenza virus demonstrated that IL-1β is responsible for the acute lung pathology [43], and the inflammasome pathway that produce IL-1β has been shown to be essential in the inflammatory response against influenza infection [7], [8], [9]. Therefore, the immunopathology seen in our acute viral infection model is likely to be related to activation of inflammasome pathway and excessive IL-1β production.

In our *in vivo* study, we found it remarkable that only a two-fold difference in the viral titer can lead to such pronounced difference in the overall host responses (Figure 6A). This result suggests that there is a threshold titer required for the induction of acute inflammation. We also observed a similar threshold effect in the *in vitro* co-culture studies where inflammasome activation and induction of the inflammatory mediators required a minimum of 20 MOI of Ad (Figure 2A, 3C, and S2). Since the induction of the inflammatory mediators was dependent on P2X_7_R, it is likely that Ad infection at the threshold titer would cause ATP release from the infected cells. Although how virus infected cells release ATP is not clear, most often ATP release is a consequence of cell damage or cell death [44]. Recently, a luciferase-based detection method was used in mice to demonstrate that extracellular ATP can accumulate at the site of inflammation to a concentration high enough to activate P2X_7_R [33], [40]. In our co-culture experiments, we detected a substantial increase in cytotoxicity, even before any significant induction of inflammatory mediators (Figure S2A). Since such cytotoxicity would potentially increase the extracellular ATP, it is conceivable that ATP released from cells dying from Ad infection would trigger the ATP-P2X_7_R mediated inflammatory responses. However, it is difficult to identify the source of ATP at the cellular level because of the instantaneous and transient nature of ATP release.

Our results from the *in vitro* study suggest that ATP is likely to be initially provided by epithelial cells as the infection of macrophages alone fails to induce inflammasome activation (Figure 3A, B and E). ATP is known to be released from stressed or injured epithelial cells [38], and infection with high dosages of Ad can induce some cell death [45]. The mechanism of macrophage activation by neighboring virus infected epithelial cells through ATP-P2X_7_R activation is in line with our previous observation that the synergistic inflammatory responses were absent when the two cells were infected in separate compartments of transwell [22]. Since P2X_7_R requires unusually high concentrations of ATP, which can be readily degraded by ectoenzymes in the extracellular space, it is believed that activation of P2X_7_R can only occur when ATP is secreted by dying cells very close to the macrophages. This mode of macrophage activation is further supported by the fact that IL-1β secretion was maximized when macrophages and epithelial cells were in 50∶50 mixtures (Fig. 3E). This co-culture condition should provide an ideal condition where ATP released from an Ad infected epithelial cell can be readily sensed by the macrophages in the vicinity.

Nevertheless, we found that the majority of the dying cells in the Ad infected co-culture were macrophages [22]. Stimulation of P2X_7_R by ATP induces inflammasome activation and release of IL-1β but an extensive exposure to ATP results in activation of pannexin-1, membrane permeablization, and eventual cell death, which would release more ATP and activate more macrophages [46]. Therefore, we propose a model (Figure S4) that ATP released from virus infected epithelial cells activates neighboring macrophages in the infected tissue, culminating in inflammasome activation and IL-1β secretion, which can further aggravate the local tissue inflammation by induction of other inflammatory mediators such as IL-6. Moreover, redundant and prolong exposure to ATP caused by persistent infection would lead to macrophage cell death. This will in turn generate more ATP for activation of macrophages and other recruited immune cells in the infected tissue, which may cause systemic inflammation through this self-amplifying mechanism.

In the experiments with ASC deficient macrophages (Raw cell line), we found that various inflammatory mediators were induced by ATP-P2X_7_R mediated signaling despite the absence of inflammasome activation. In particular, we found that NO (Figure 2A) and ROS (Figure 2C) generation was completely dependent on P2X_7_R as previously reported [27]. These results indicate that ATP-P2X_7_R signaling may regulate various cellular responses in addition to the inflammasome activation. Since the mechanism downstream of ATP-P2X_7_R leading to the inflammasome activation is not clearly understood, it would be of interest to explore how ATP triggers induction of these important inflammatory mediators in cells lacking inflammasome components. In this regard, it is noteworthy that Ad infection induced ROS in Raw cells (Figure 1B) whereas the inhibition or deficiency of P2X_7_R did not produce ROS (Figure 1B and 2C). Currently, there is a controversy about whether inflammasome activation is mediated by ATP or ROS [12]. Although it requires further analyses our data indicate that ATP-P2X_7_R activation might be at the upstream of ROS generation.

While production of NO and ROS was completely dependent on P2X_7_R in the MLE-Raw co-culture (Figure 1 and 2), IL-1β secretion and inflammasome activation in MLE-J774 co-culture was partially inhibited by the deficiency of P2X_7_R and caspase-1 (Figure 4 and 5). Moreover, P2X_7_R and caspase-1 deficiency protect 40% of mice while others succumbed to ARDS in our *in vivo* study (Figure 6). Similar results were obtained from previous studies that examined the role of P2X_7_R in systemic inflammation in a graft-versus-host disease model [33].The reason for the partial inhibition might be due to the redundancies and complex network of interrelated pathways in innate immune system [16]. For instance, other purinergic receptors like P2Y_2_R are known to provide ATP mediate inflammation in recruiting neutrophils [47]. In addition, other cytokines may be able to compensate for the reduced of IL-1β secretion. In particular, IL-1α, which binds to the same receptor and induces the same inflammatory responses as IL-1β, does not require post-translational modification through inflammasome pathway for its biological function [42]. Considering these redundancies and compensating mechanisms, the significant difference in the survival rate underscores the importance of ATP-P2X_7_R mediated inflammatory responses.

Taken together, our findings support a model that a high concentration of ATP released from virus infected cells during an acute viral infection functions as a danger signal, which activates the inflammatory responses including inflammasome pathway and may serve as a link between the local infection and systemic inflammation (Figure S4). The results of this study indicates that controlling excessive inflammation by inhibiting ATP-P2X_7_R mediated signaling pathway could provide a possible therapeutic approach for diseases caused by acute inflammation. Although such therapeutic approach could provide beneficial effects in many conditions in the absence of infective agents, it should be noted that disruption of an innate immune mechanisms can also compromise pathogen clearance and increase fatality as seen in infections with wild-type viruses [12], [13]. Therefore, the therapy should consider measures to control both pathogen and ATP induced inflammatory responses.

## Materials and Methods

### Ethics Statement

All animal use procedures were conducted according to the guideline set by the Canadian Council on Animal Care. The Animal Care Committee at the Hospital for Sick Children, approved all protocols developed for this work.

### Reagents

LPS, oATP, apyrase were purchased from Sigma (St. Louis, MO, USA), z-YVAD-fmk from BioVision (Mountain View, CA, USA), and A-438079 from Tocris (Ellisville, MO, USA).

### Adenovirus vectors

The Ads used in this study were prepared as described previously [22], [48]. The helper-dependent Ad was used in the *in vitro* experiments and the E1-deleted first generation Ad was used in the *in vivo* studies in order to achieve the high viral dosages required for the experiment.

### Cell culture and *in vitro* infection

The cell lines and the primary macrophage cells were cultured in DMEM (Invitrogen, Burlington, ON, Canada) supplemented with 10% FBS (Invitrogen) and cultured at 37°C in 5% CO_2_. The macrophage and epithelial cell co-cultures were established and infected with Ad as previously described [22]. Briefly, MLE and macrophage (Raw, SF, J774, ATPR, or peritoneal macrophages) co-culture was prepared on 6 well plates by first seeding MLE cells and the macrophages on the following day, each at 25% confluency. On the third day the co-culture was about 80∼90% confluent with MLE and macrophages at 50∶50 ratio. The mono-cultures of MLE or macrophages were also grown to 80∼90% confluency before Ad infection. The cell culture was replaced with 1 ml of fresh medium and Ad was directly inoculated to the medium at approximately 20 multiplicity of infection (MOI). The mouse primary peritoneal macrophages were collected by peritoneal lavage as previously described [10] except lavage was performed without the thioglycollate induction.

### Measuring inflammatory mediators and cytotoxicity

Analyses of inflammatory mediators and cytotoxicity were performed as previously described [22]. Briefly, IL-6 and KC were measured from the culture medium using available ELISA kits (R&D Systems, Minneapolis, MN, USA) and NO was measured by Griess assay (Promega, Madison, WI, USA). The ROS positive cells were analyzed by flow cytometry using 3-(p-aminophenyl) fluorescein (APF; Invitrogen, Burlington, ON, Canada). The cytotoxicity in the culture was assessed by using a LDH assay kit (Promega, Madison, WI, USA). Commercially available ELISA kits were used to measure IL-1β (BD Biosciences, Mississauga, ON, Canada) and IL-18 (MBL, Nagoya, Japan). IL-1β was detected by Western blot analysis following immuno-precipitation with an antibody against mouse IL-1β (R&D Systems, Minneapolis, MN, USA).

### *In vivo* Ad infection

C57Bl/6 mice (Charles River, St. Constant, QC, Canada), caspase-1-KO (gift from Dr. Alberto Martin), and P2X_7_-KO mice (Jackson Laboratory, Bar Harbor, ME, USA) were used for *in vivo* experiments. Mice 6∼10 weeks of age were briefly anesthetized by isoflurane inhalation and 50 µl of Ad solution containing 1×10^11^ of viral particles was administered through nares into the lungs. The inhibitors were administered by intraperitoneal injection for the first two days of infection. BALF was obtained as described elsewhere [32] and used for cytokine measurement and differential cell count. Mice with more than 20% weight loss were euthanized.

### Histology

The mouse lung sections were prepared from the whole lung samples fixed in 4% buffered formaldehyde. The sections were stained with hematoxylin and eosin and the severity of pathological features were assessed according to the criteria described in the Supplementary Material (Table S1).

### Differential cell count

The cell fraction from the BALF were prepared on a slide by cytospin and differential cell counts were performed in a blinded manner, with a total of 300 cells counted per sample.

### Statistical analyses

Student's t-test was used to assess statistical significance between means. Survival curves were compared by using the log rank test. Significance (*) was set at p<0.05. The error bar represents mean ± standard deviation (SD) of three independent experiments.

## Supporting Information

Figure S1 **Ad infection of MLE-Raw co-culture induced IL-1β but fail to process it to mature form.** The Raw cell mono-culture and MLE-Raw co-culture were infected with Ad (20 MOI) for 18 h and cells were harvested for Western blot analysis. Proteins were resolved in 15% SDS-PAGE, and IL-1β was detected by an anti-mouse IL-1β antibody (R&D Systems, Minneapolis, MN, USA), which detects both pro-IL-1β (p-IL-1β) and mature IL-1β (m-IL-1β).
(TIF)

Figure S2 **Ad infection of MLE-J774 co-culture increased cytotoxicity and induced pro-inflammatory cytokines.** The MLE-J774 co-culture was infected with the designated doses (MOI) of Ad. (A) The cytotoxicity was measured by LDH assay using the culture media 18 h after infection. The increase in cytotoxicity was presented as the relative fold increase compared to the non-infected culture. (B and C) The induction of pro-inflammatory cytokines, IL-6 (B) and KC (C), were measured by ELISA using the media obtained from MLE-J774 co-culture 24 h after infection with different doses of Ad. Data are expressed as mean ± SD.
(TIF)

Figure S3 **The survival advantage of P2X_7_R-KO mice is not due to better lung histopathology.** The wild-type (C57/B6) and the P2X_7_R KO mice were infected with Ad of 1×10^11^ vp/mouse by i.n. administration. The mice were sacrificed on day 2 (n = 3), or day 6 (n = 3) after administration and the lungs were obtained for histological analysis. There were no significant differences between the wild-type and the P2X_7_R KO mice. Representative microscopic sections are shown. See also Figure 7A and B for sections from day 4 and the lung histology score analysis.
(TIF)

Figure S4 **Model of ATP regulating inflammatory response during viral infection.** A viral infection is sensed by macrophages via various pathogen recognition receptors (PRRs) on the plasma membrane and in the cytoplasm. Sensing of viral infection activates NFκ-B and other inflammatory signaling pathways to induce pro-inflammatory cytokines (Signal 1). As one of the key pro-inflammatory cytokines, IL-1β is produced in the inactive form of pro-IL-1β. Acute viral infection can cause release of a high concentration of ATP by the infected epithelial cells. The released ATP is sensed by P2X_7_R on the cell membrane and induces channel activation and potassium ion efflux in macrophages (Signal 2). These changes allow inflammasome formation and activation of caspase-1, which cleaves pro-IL-1β to active IL-1β. IL-1β is then secreted from the cell to induce various inflammatory responses. An extensive activation of P2X_7_R causes pennexin-1 activation, membrane permeablization, and eventual macrophage cell death, which leads to release of more ATP and other danger signals. These self-amplifying events would lead to escalation of inflammatory responses and may result in systemic inflammation.
(TIF)

Table S1 The criteria for assessing mouse lung pathology score.
(DOC)
