# Supplementary figures and images for: Activation of P2X7 Receptor by ATP Plays an Important Role in Regulating Inflammatory Responses during Acute Viral Infection
Source: PLoS One. 2012 Apr 25;7(4):e35812. doi: 10.1371/journal.pone.0035812 (PMC3338466; doi:10.1371/journal.pone.0035812)

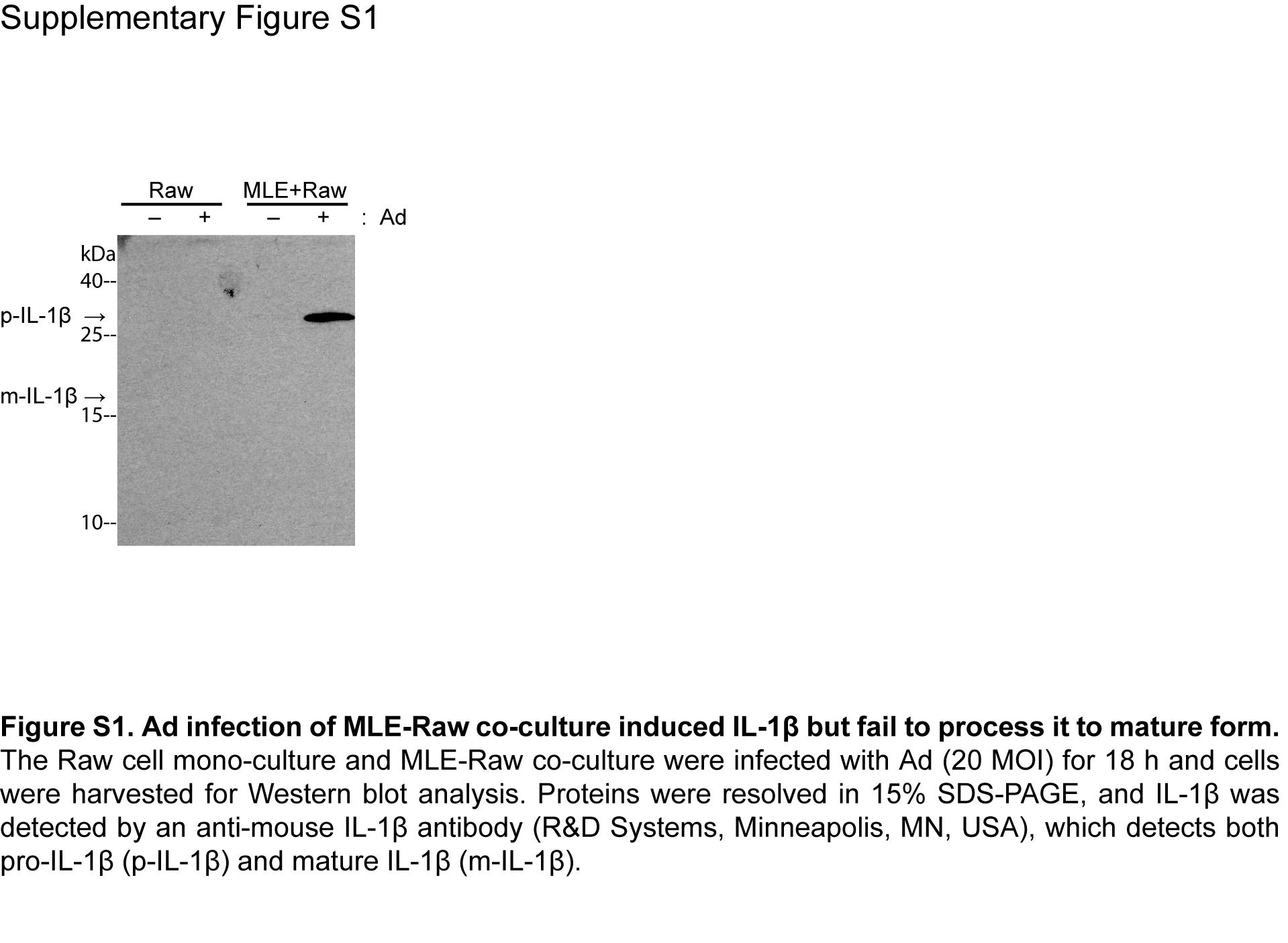

Supplement: Figure S1 — Ad infection of MLE-Raw co-culture induced IL-1β but fail to process it to mature form. The Raw cell mono-culture and MLE-Raw co-culture were infected with Ad (20 MOI) for 18 h and cells were harvested for Western blot analysis. Proteins were resolved in 15% SDS-PAGE, and IL-1β was detected by an anti-mouse IL-1β antibody (R&D Systems, Minneapolis, MN, USA), which detects both pro-IL-1β (p-IL-1β) and mature IL-1β (m-IL-1β). (TIF) [file pone.0035812.s001.tif]

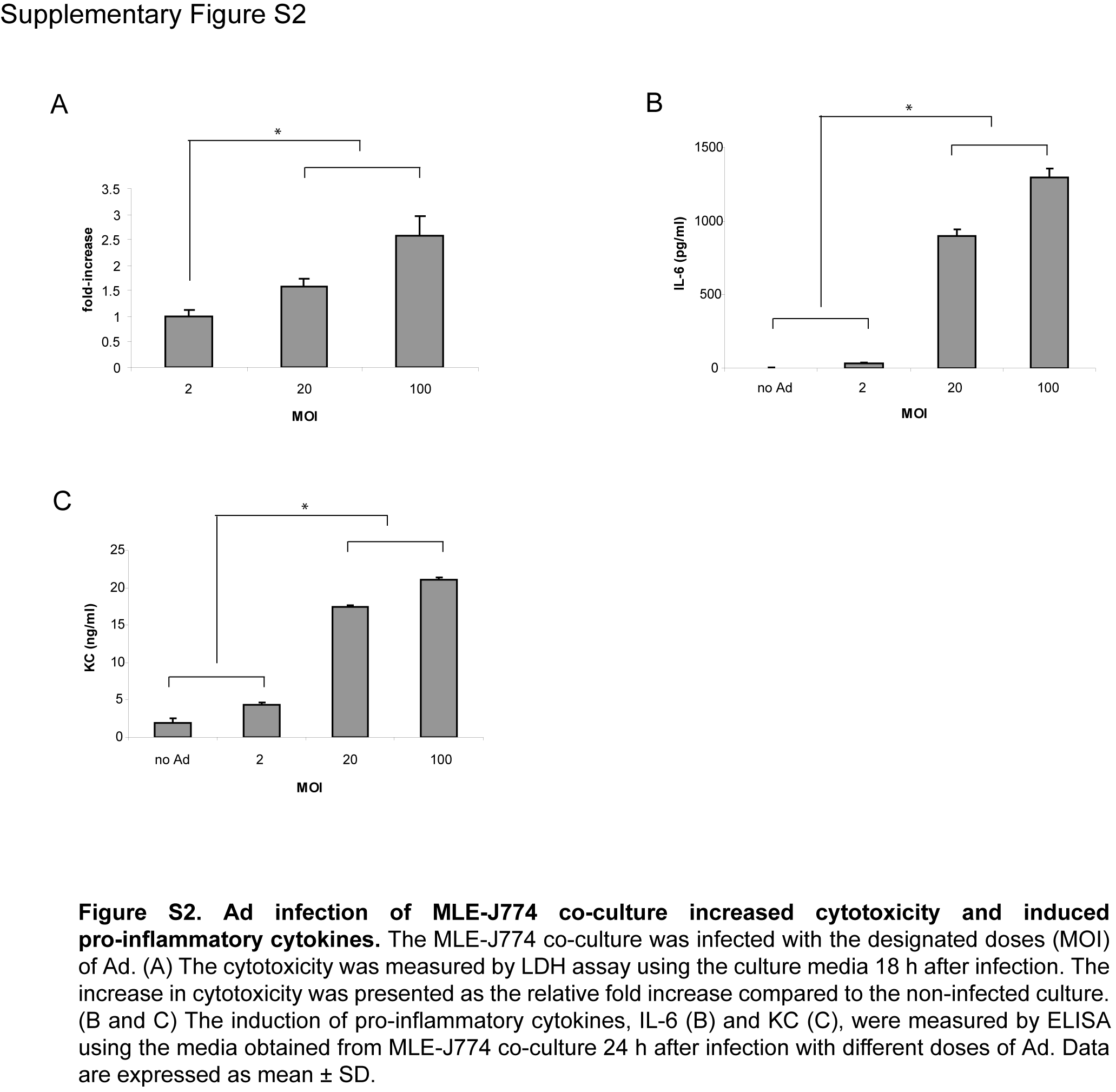

Supplement: Figure S2 — Ad infection of MLE-J774 co-culture increased cytotoxicity and induced pro-inflammatory cytokines. The MLE-J774 co-culture was infected with the designated doses (MOI) of Ad. (A) The cytotoxicity was measured by LDH assay using the culture media 18 h after infection. The increase in cytotoxicity was presented as the relative fold increase compared to the non-infected culture. (B and C) The induction of pro-inflammatory cytokines, IL-6 (B) and KC (C), were measured by ELISA using the media obtained from MLE-J774 co-culture 24 h after infection with different doses of Ad. Data are expressed as mean ± SD. (TIF) [file pone.0035812.s002.tif]

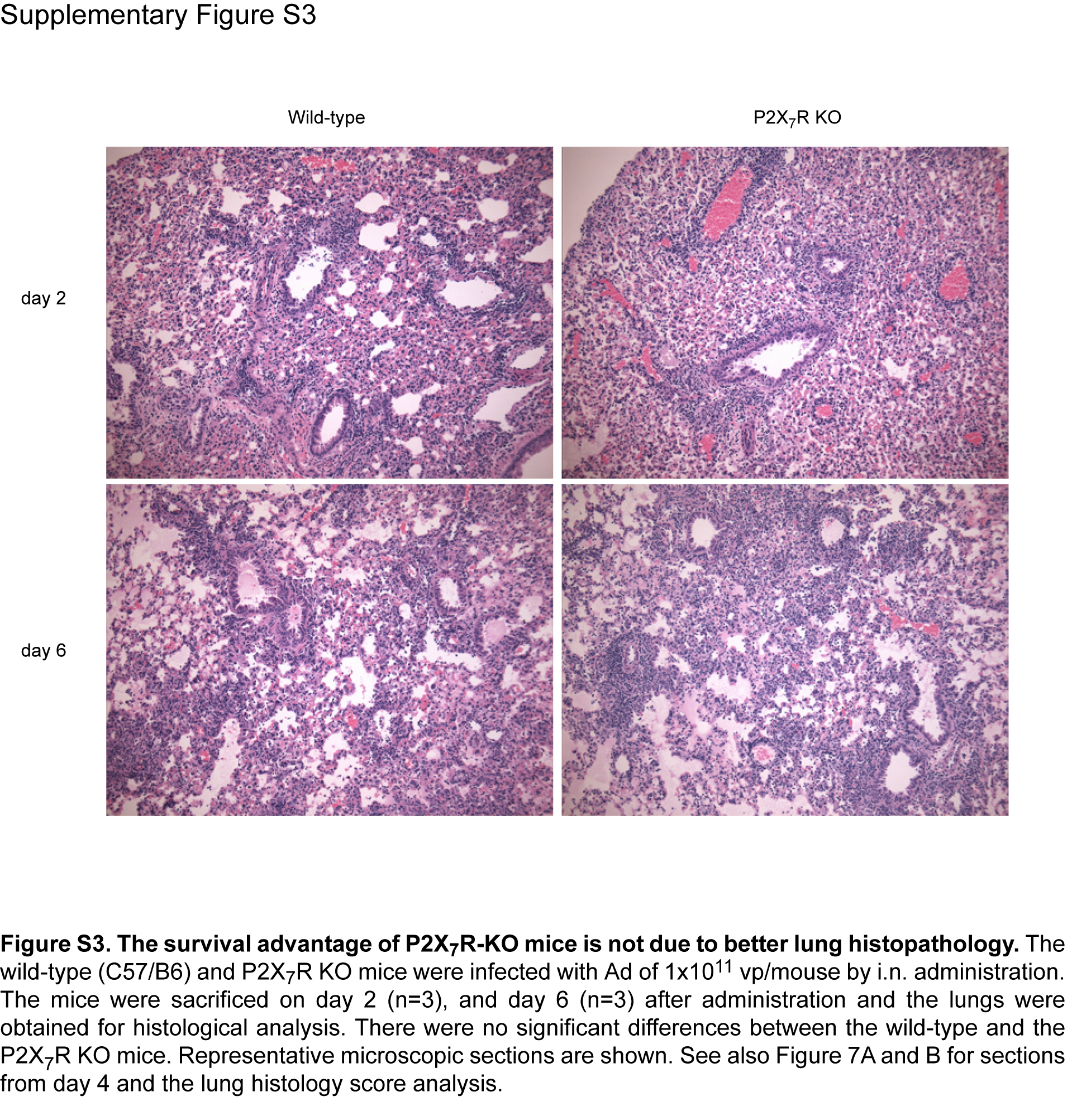

Supplement: Figure S3 — The survival advantage of P2X7R-KO mice is not due to better lung histopathology. The wild-type (C57/B6) and the P2X7R KO mice were infected with Ad of 1×10^11^ vp/mouse by i.n. administration. The mice were sacrificed on day 2 (n = 3), or day 6 (n = 3) after administration and the lungs were obtained for histological analysis. There were no significant differences between the wild-type and the P2X7R KO mice. Representative microscopic sections are shown. See also Figure 7A and B for sections from day 4 and the lung histology score analysis. (TIF) [file pone.0035812.s003.tif]

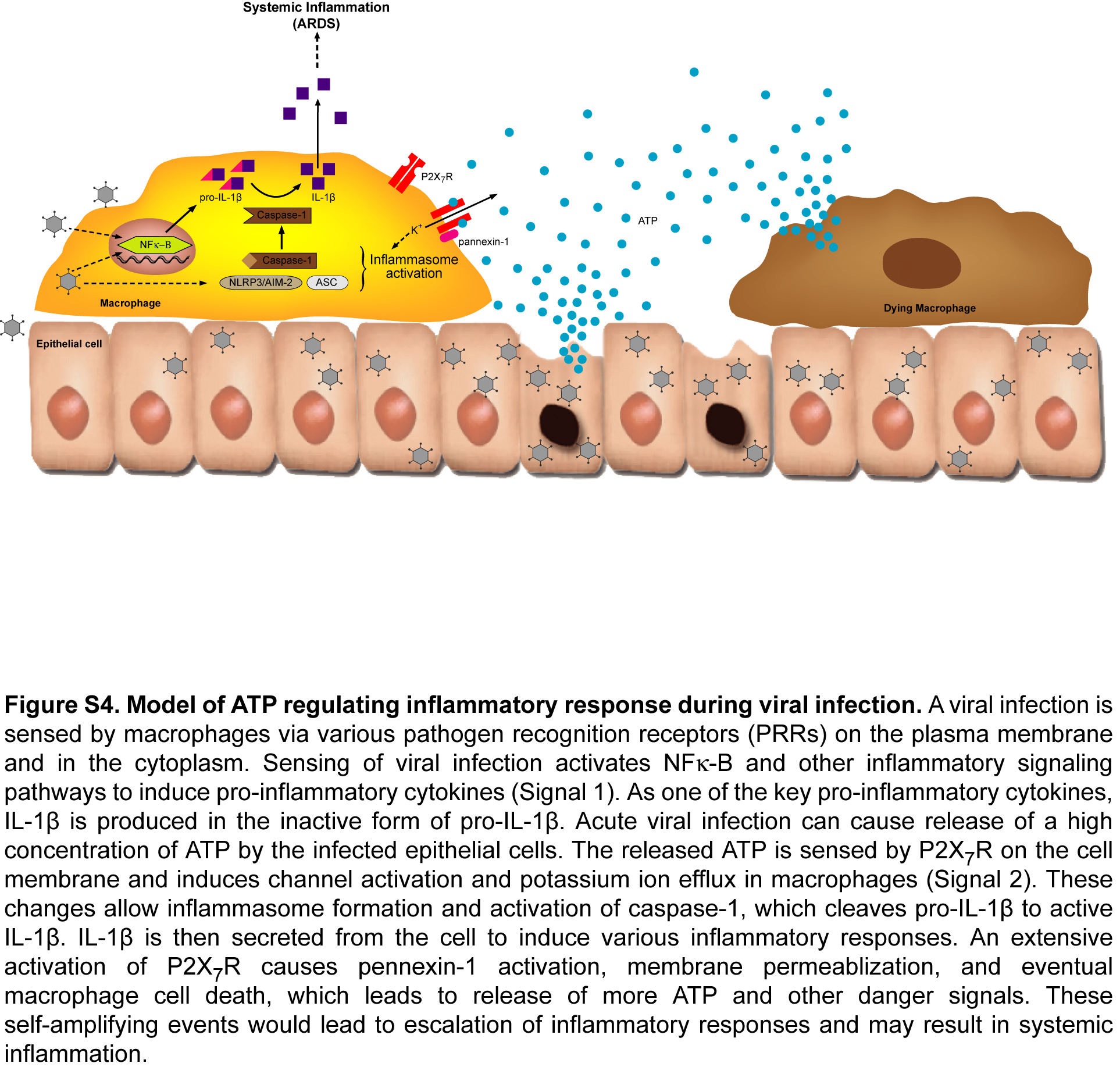

Supplement: Figure S4 — Model of ATP regulating inflammatory response during viral infection. A viral infection is sensed by macrophages via various pathogen recognition receptors (PRRs) on the plasma membrane and in the cytoplasm. Sensing of viral infection activates NFκ-B and other inflammatory signaling pathways to induce pro-inflammatory cytokines (Signal 1). As one of the key pro-inflammatory cytokines, IL-1β is produced in the inactive form of pro-IL-1β. Acute viral infection can cause release of a high concentration of ATP by the infected epithelial cells. The released ATP is sensed by P2X7R on the cell membrane and induces channel activation and potassium ion efflux in macrophages (Signal 2). These changes allow inflammasome formation and activation of caspase-1, which cleaves pro-IL-1β to active IL-1β. IL-1β is then secreted from the cell to induce various inflammatory responses. An extensive activation of P2X7R causes pennexin-1 activation, membrane permeablization, and eventual macrophage cell death, which leads to release of more ATP and other danger signals. These self-amplifying events would lead to escalation of inflammatory responses and may result in systemic inflammation. (TIF) [file pone.0035812.s004.tif]
