# Supplementary material for: Activation of P2X7 Receptor by ATP Plays an Important Role in Regulating Inflammatory Responses during Acute Viral Infection
Source: PLoS One. 2012 Apr 25;7(4):e35812. doi: 10.1371/journal.pone.0035812 (PMC3338466; doi:10.1371/journal.pone.0035812)
Supplement: Table S1 — The criteria for assessing mouse lung pathology score. (DOC) [file pone.0035812.s005.doc]

**Table S1**. The criteria for assessing mouse lung pathology score.

| **Criteria** | **Pathology score** | | | |
| --- | --- | --- | --- | --- |
|  | **0** | **1** | **2** | **3** |
| Overall extent of inflammation | none | mild  (<10% of lung area affected) | moderate  (10-25% of lung area affected) | severe  (>25% of lung area affected) |
| Airway inflammation/injury | none | mild  (scattered bronchial/peribronchial inflammation without intraepithelial infiltration, epithelial cell apoptosis or necrosis; +/- focal reactive epithelial changes) | moderate  (band-like bronchial/peribronchial inflammation with widespread epithelial reactive/reparative changes; +/- focal epithelial cell apoptosis) | severe  (inflammation with epithelial necrosis or widespread apoptosis) |
| Alveolar inflammation/injury | none | mild  (scattered alveolar septal inflammation without significant reactive pneumocyte hyperplasia) | moderate  (alveolar septal inflammation with patchy edema and reactive pneumocyte hyperplasia/atypia) | severe  (alveolar septal inflammation with widespread edema, intra-alveolar fibrinous exudates/hyaline membranes, prominent reactive pneumocyte changes) |
